# Location Estimation in a Smart Home: System Implementation and Evaluation Using Experimental Data

**DOI:** 10.1155/2008/142803

**Published:** 2008-04-16

**Authors:** Youcef Rahal, Hélène Pigot, Philippe Mabilleau

**Affiliations:** Laboratoire DOMUS, Université de Sherbrooke, 2500 boulevard de l'Université, Sherbrooke, QC, Canada J1K 2R1

## Abstract

In the context of a constantly increasing aging population with cognitive deficiencies, insuring the autonomy of the elders at home becomes a priority. The DOMUS laboratory is addressing this issue by conceiving a smart home which can both assist people and preserve their quality of life. Obviously, the ability to monitor properly the occupant's activities and thus provide the pertinent assistance depends highly on location information inside the smart home. This paper proposes a solution to localize the occupant thanks to Bayesian filtering and a set of anonymous sensors disseminated throughout the house. The localization system is designed for a single person inside the house. It could however be used in conjunction with other localization systems in case more people are present. Our solution is functional in real conditions. We conceived an experiment to estimate precisely its accuracy and evaluate its robustness. The experiment consists of a scenario of daily routine meant to maximize the occupant's motion in meaningful activities. It was performed by 14 subjects, one subject at a time. The results are satisfactory: the system's accuracy exceeds 85% and is independent of the occupant's profile. The system works in real time and behaves well in presence of noise.

## 1. INTRODUCTION

The outburst of aging population in the recent and forthcoming years lays new challenges to provide assistance to the elders. Moreover, many elders may present degenerative diseases in their later years which can affect their cognitive abilities. For example, in Canada [1], people older than 65 will represent at least 25% of the population in 2030, and in 2021 the number of patients with Alzheimer and other related diseases will reach 592 000 persons (compared to 364 000 in 1992). Therefore, it becomes urgent to find a compromise between the need for constant care (at home or in institutions) and the need to lighten the load on caregivers. Also, it is highly important to ensure that the ministered care is personalized and efficient. In this regard, the Smart Assistive Home concept is an adequate solution. Indeed, it is now possible to provide a safe environment where the occupants (elders with cognitive deficiencies) can both be autonomous and feel familiar. The smart home can also be a good alternative to people who suffered cranial trauma. This population is generally young and benefits from less specialized institutions than the elders.

The DOMUS laboratory, presented in paper [2], plans to address the above issues by conceiving and testing a smart home. It is located at Université de Sherbrooke, Canada. The experimental apartment consists of a bedroom, a bathroom, a living room, a dining room, and a kitchen. The house is filled with different kinds of sensors in order to provide an accurate information about the occupant's location and activities. It can also interact with the occupant via different effectors such as touch screens, audio speakers, and controllable lights. Robust user-system interaction is ensured thanks to pervasive computing.

The occupant's location is probably one of the most important data needed to monitor the occupant's activities. Indeed, this information is useful when interacting with the occupant and in preventing dangers (by detecting falls, e.g.,). In the context of DOMUS, this information is vital in order to infer the activities already performed or those being processed, and to provide assistance where the occupant is. Our goal is to build a robust and accurate localization system using the available set of sensors already installed in the smart home. It covers the case when a single occupant is present at home, which is the most relevant case when assisting people with cognitive deficiencies. We first analyze the current localization techniques regarding certain constraints. We then present the formalism of the method we selected and the experimental setup. Finally, we present the experiment we conducted, meant to thoroughly evaluate our system, and we discuss the results obtained. The current paper extends [3], focuses on evaluation, and thus completes the corresponding results and discussion sections. We do not discuss herein higher levels of assistance meant to detect falls, infer current activities, or interact with the occupant, and which can be implemented using the information provided by our localization system.

## 2. TECHNIQUES AND CONSTRAINTS

Only a few AI and robotics localization techniques can be applied in a small-scale environment [4]. Moreover, depending on the experimental constraints, the amount of available solutions is drastically reduced. These constraints are derived from two principles applied within the DOMUS technology. First, the occupant's privacy must be guaranteed. Second, the technology must be unobtrusive. This leads to the four following constraints.

- The use of video cameras is prohibited in order to protect the occupant's privacy.
- The sensors should be dissimulated in the house to provide a familiar environment.
- The use of tags worn by the occupant is avoided. This reduces anxiety and the occupant's feeling of being constantly monitored.
- The most economic solutions are preferred.

The first constraint rejects video localization systems such as the one described in [5]. Considering the third constraint, solutions such as radio frequency identification (RFID) tags [6] or Wi-Fi engines [7] are also pushed aside. This is also the case with infrared (IR) or ultrasonic badges [8]. The cost constraint eliminates floor-based sensors, as described in [9]. In fact, this leaves us with inexpensive solutions which collect anonymous data. This includes devices such as IR detectors and a few other sensors which are already installed in the DOMUS home. Even in quantity, a system based on such sensors is quite affordable compared to the aforementioned solutions. We discuss the full list in Section 4.1.

Localization errors are induced when relying directly on the sensors, because the latter may sometimes send false information. This may happen because of an intrinsic error, which can be due to the sensor's error rate or to an occasional error in the experimental setup as a whole. External factors can also cause false sensor information. For instance, a draft can close a door and thus trigger a false event. So can pets sometimes. Therefore, the reliability of the localization system depends on our ability to analyze the sensor data. In this regard, recent researches show that sensor fusion is an efficient way to reinforce the validity of the location data. Whether in robotics [10] or in indoors localization [11], sensor fusion is achieved through probabilistic methods such as Bayesian filtering.

## 3. PARTICLE FILTERS

Bayes filters are efficiently used to estimate a person's location using a set of fixed sensors. In this method, the last known position and the last sensor event are both used to estimate a new location. The method represents an interesting compromise between accuracy and performance, and can be implemented in different ways. Fox et al. describe a few techniques to implement Bayesian filters and compare their performances [11]. Based on the results of that comparison, we decided to implement a localization system using the particle filters approach. Indeed, Kalman filters is an accurate method. However, it becomes inappropriate when different types of sensors are considered. This is the same for multihypothesis tracking. Also, grid-based approaches are robust but their poor efficiency excludes them. Finally, topological approaches do not meet the criteria, except for the robustness. On the other hand, particle filters are an efficient technique which is accurate, robust, and easy to implement. This technique is also adequate when different types of sensors are used, which is our case. We will briefly describe the technique. Refer to [11, 12] for further reading.

At any moment, the estimate of the occupant's location can be modeled as the belief in the fact that he/she is located at position *x_t_* at instant *t*, given a series of previous sensor observations *o*_0_,…,*o_t_* from instant 0 to instant *t* : Bel (*x_t_*) = *p*(*x_t_* ∣ *o_t_*, *o*_*t*−1_,…,*o*_1_,*o*_0_). Given the Markov postulate which stipulates that only the last observation *o*_*t*−1_ is relevant, the expression of Bel(*x_t_*) is simplified using Bayes conditional probabilities formula. In the discrete case, it becomes

(1) $$\text{Bel}\;(x_{t})=\alpha p\;(o_{t}\;|\;x_{t})\underset{x_{t-1}}{\sum }p\;(x_{t}\;|\;x_{t-1})\;\text{Bel}\;(x_{t-1}),$$

where *α* is a normalization constant. *p*(*o_t_* ∣ *x_t_*) represents the probability of observing an event *o_t_* given that the occupant is at position *x_t_* at instant *t*. It is the perceptual model. In practice, each sensor is associated to a space-dependent probability density function that represents the likelihood of receiving an event from that sensor depending on the occupant location. On the other hand, *p*(*x_t_* ∣ *x*_*t*−1_) represents the probability that the occupant moves from *x*_*t*−1_ to *x_t_* between instants *t* − 1 and *t*. It is the dynamic model. In practice, it represents the motion profile of the occupant. For instance, a fast-moving occupant has a wider dynamic probability density than a slower one. This function should also include the environmental layout. In a more rigorous approach, it should even depend on time to reflect the changes in activities during the day or the week.

The algorithm based on particle filters estimates the location thanks to a set of *n* positions (particles). At first, these particles are drawn randomly and uniformly on the available space. An equal weight is devoted to each particle (1/*n* for the sake of normalization). These particles model Bel(*x*_0_) which, in this case, is uniform. When an event occurs (at *t* = 1), Bel(*x*_1_) is computed according to (1). This operation changes the weights of the sample. The distribution obtained is then used to draw a new sample with equal weights. The new sample is from now on more centered on the latest event position. This operation is repeated every time a new event is observed. At any time, the estimate (belief) of the occupant's position in a place is simply the addition of the weights of all the particles confined in that location. Consequently, we can observe in practice a cloud of particles “following” the occupant.

Before using this technique for location estimation in the smart home, we need to make an inventory of all the sensors we use and then attribute probability density functions to them. We also need to model the occupant's motion. The next section deals with these aspects.

## 4. EXPERIMENTAL CONFIGURATION

### 4.1. The sensors

The list of sensors we consider and which are already installed and plugged in the DOMUS apartment include the following.

- IR presence detectors.
- Tactile carpets (as seen later in Section 6.1, for the purpose of this study, these sensors are used only as location reference and not to infer the occupant's position).
- Smart light switches. An event is received every time the occupant turns the lights on or off.
- Electric contacts on doors (including closets, drawers, pantries, etc.). An event is received every time a door is opened or closed.
- Pressure detectors. These can be placed under the mattress, for example, in order to detect if the occupant is lying on the bed.

The number of installed sensors varies depending on the room and the areas of interest (see Table 1). For example, in the bedroom there is only one IR detector that covers the entire room area whereas two are installed in the kitchen: one covers the kitchen globally while the other is directed at the stove only. It is worth noting that none of these sensors would be able to give identification about the person who triggered it—as opposed to devices such as video cameras. They are also quite unobtrusive. That is clearly an advantage of our approach since it provides the required privacy imposed by our constraints.

### 4.2. Probability densities

We assign a probability density function to each sensor. This function depends on the sensor type and, mainly, on the range the sensor covers. For instance, IR detectors have a wide probability density, taking into account the fact that they cover large portions of a room (of course, this depends on how they are installed; they can also be installed to cover small regions). Moreover, they can be triggered more easily than the other sensors. Light switch sensors are generally more reliable and are triggered in a limited area. So are the door contacts, the tactile carpets, and the pressure detectors. That is why we assigned to them more compact probability densities than to IR detectors. We list the attributes of these functions in Table 2. These functions are either 2D square functions defined as

(2) $$p\;(x,\;y)=\{\begin{array}{ll} \frac{1}{\left(x_{\text{max}}\;-\;x_{\text{min}})(y_{\text{max}}\;-\;y_{\text{min}}\right)}, & x_{\text{min}}<x<x_{\text{max}},\\ & y_{\text{min}}<y<y_{\text{max}},\\ 0, & \text{elsewhere,} \end{array}$$

or 2D circular functions as

(3) $$p\;(x,\;y)=\left\{ \begin{array}{ll} \frac{1}{\pi R^{2}}, & {\left(x-x_{0}\right)}^{2}+{\left(y-y_{0}\right)}^{2}<R^{2},\\ 0, & \text{elsewhere}\text{.} \end{array}\right.$$

We do not add noise to these functions per se. However, we make sure that, at every step, about 10% of the particles are drawn randomly from all the available space. We noticed that adding noise to density functions could result in a static particle cloud after successive sensor events in the same place. In other words, the cloud can converge in a relatively small area and remains there regardless of new sensor events. By adding the uniform noise, we ensure that there are enough particles in the environment to avoid the cloud's immobilization. As for the dynamics, the occupant's motion is modeled by a 2D normal density which standard deviation encompasses half of the apartment's area. Although this function is quite rough, it models remarkably well the fact that, between instants *t* − 1 and *t*, the likelihood that the occupant covers a distance *d* quickly decreases when *d* increases.

## 5. IMPLEMENTATION

**Figure 1:**
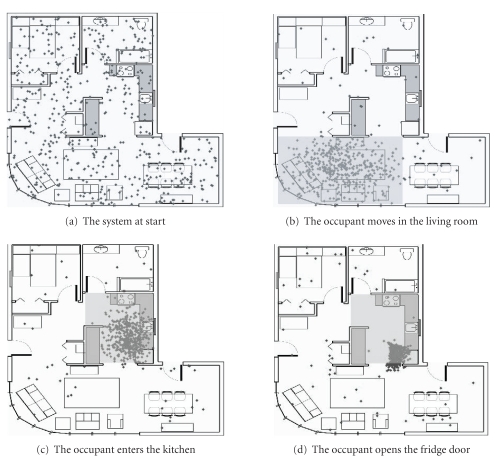
The localization system in a real condition situation. The points show the particle cloud. The shaded areas indicate where the occupant is likely to be present.

Our system is implemented in Java and connects as a client to a server which aggregates sensors and forwards events as soon as they occur. We also implemented a GUI that displays the client's state and the current prediction of the system. Figure 1 shows typical use of our system in real conditions. These are based on the observation of its behavior during the execution of a short scenario, similar to the one we describe in Section 6.1. We perform the scenario and we observe the system's predictions, knowing the occupant's location. In Figure 1(a), we start the system. The occupant is already in the living room. However, since no sensor event is detected yet, the occupant is likely to be anywhere in the house. Consequently, the particle cloud is uniformly spread. In Figure 1(b), the occupant moves towards the kitchen and the living room IR sensor is activated. The system consequently computes that the occupant is in the living room with a probability of 91%. The particle cloud is contained in the corresponding area. In Figure 1(c), the occupant enters the kitchen and the IR sensor there is activated. The system concludes that the occupant is in the kitchen at 91%. Finally, the occupant opens the fridge door and a door-contact event is received. The cloud is quickly centered on the corresponding area. Here also, the probability that the occupant is in the kitchen is high: 81%. The probability is less than in the previous case because the probability density function (the perceptual model) associated to the fridge's door-contact sensor encompasses a small zone outside the kitchen. The particle cloud is very dynamic and smoothly follows the occupant's path. The computation time (the one needed to infer the occupant's location when receiving a new event) is inferior to 1 second and thus the system is responsive in real time.

## 6. SYSTEM EVALUATION

While the preliminary results are encouraging, it is important to thoroughly evaluate the system's accuracy and robustness. It becomes then necessary to monitor how it behaves in real-life situations. Therefore, we conceived an experiment to collect data with people moving inside the apartment, one person at a time. The subjects have no prior familiarity with the apartment.

### 6.1. The experiment scenario

Each subject performs a scenario of about 50 minutes long. It consists of the contracted routine of getting back to home in the evening and leaving in the following morning. This scenario maximizes the occupant's motion in every room of the apartment, all the while allowing to perform meaningful activities. It also maximizes the number of activated sensors during tasks execution. This ensures that our system is able to locate the person with accuracy in real conditions. The broad lines of the scenario are

1. entering the house;
2. washing hands in the bathroom;
3. preparing a sandwich in the kitchen;
4. eating the sandwich in the dining room;
5. preparing coffee in the kitchen;
6. reading a magazine in the living room while drinking coffee;
7. going to the bathroom;
8. lying in the bedroom (the subject is allowed to read while being on the bed);
9. getting up and making toilette in the bathroom;
10. leaving the house.

To avoid cognitive load, we ask the subjects to perform this scenario by periods of about 10 minutes each. For example, the three first steps of the scenario fit into such a period. At the end of each period, we stop the data collection and explain the next set of tasks. We validated the entire scenario during a preexperimentation with 3 team members. This helped us adjust the steps and make the scenario more fluent.

The accuracy of the location estimation is checked thanks to a video camera and the tactile carpets. The camera is located in the kitchen and is used to record the subject's activities during the preparation of the sandwich. Five tactile carpets are installed on the kitchen's floor. The analysis of the video validates the tactile carpets accuracy (since we use the tactile carpets in the framework of a complex acquisition system, the accuracy we measure is not rigorously equal to the one we would obtain if the carpets were used separately from this system). Knowing this accuracy, we use the tactile carpets in every room of the apartment as reliable position indicators. We compare this reference position information with the output of our system. This gives the system's accuracy, that is, the accuracy of the location information resulting of Bayesian filtering using the rest of the sensors.

### 6.2. The sample

**Figure 2:**
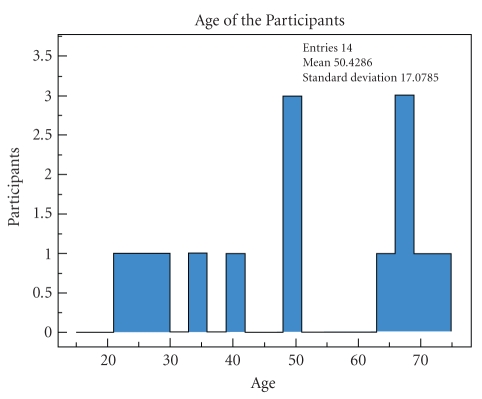
Distribution of the age of the subjects.

The sample of the 14 subjects who participated in the experiment is composed of 10 females and 4 males. Their age distribution is shown on Figure 2. The age is well distributed between 22 and 73, with a mean value of 50 years old. There is however a slight majority of people older than 50. To reflect the population targeted by the research at DOMUS, we recruited at least half of the subjects from that age group.

## 7. RESULTS AND DISCUSSION

**Figure 3:**
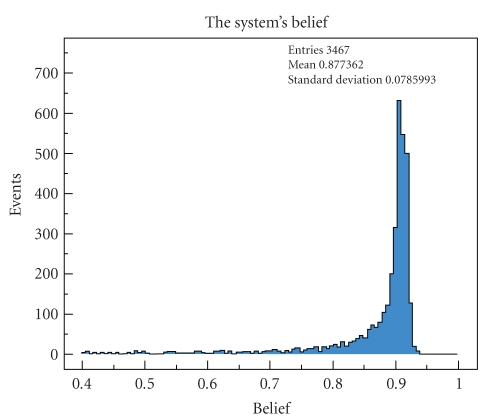
Distribution of the belief of our system, regrouping the data from the whole sample.

First of all, we observe the distribution of the system's belief. As shown in Figure 3, the mean belief is 88% with a standard deviation of 8% for all the data from our sample. There is an upper limit to the system's belief which is close to 90%. It is due to the 10% of noise particles that we draw randomly at every iteration. These are uniformly distributed in the smart home, and therefore the concentration of particles in a room cannot significantly exceed 90%.

**Figure 4:**
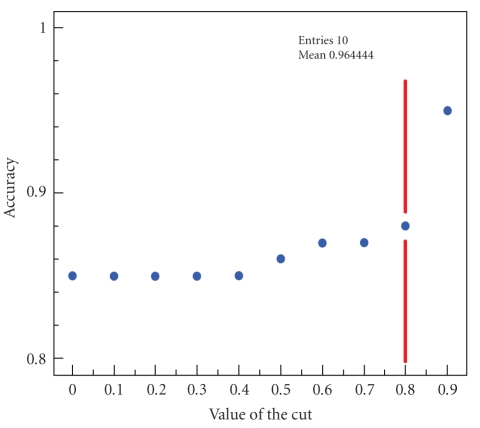
Variation of the accuracy depending on the value of the rejection cut on the system's belief.

The events with a low belief usually occur when the occupant goes from a room to another. With the following events, the belief gets significantly higher, showing that the particle cloud replaces itself quickly and correctly. Moreover, the global system's accuracy is 85%. This is the percentage of events where the system predicts accurately the position of the occupant, independently from the value of the system's belief. There is however an expected correlation between the system's accuracy and its belief. Events with a high belief are less prone to be false, and vice versa. We can therefore increase the system's accuracy by rejecting the events with the smallest beliefs. Figure 4 shows the variation of accuracy with the value of the rejection cut on the system's belief. By rejecting events with belief less than 80%, the accuracy of the system becomes 88%. This cut rejects only 6% of true events and up to a third of false ones. The cut can of course be more drastic: by rejecting events with a belief less than 90%, accuracy can be increased to 95%. However, the cut at 80% is more reasonable, since this value is the mean belief for false events. In working conditions, one can vary the cut depending on how critical the location information has to be, that is, depending on the context and on the application that needs the information.

The most important limitation is that, as expected, our system gives incoherent results in case more than one person are present in the house. Indeed, when two people (or more) activate sensors simultaneously, the particle cloud tends to alternate between their respective locations. An upper-level solution is then necessary in order to identify and handle this trend. This limitation is a direct consequence of the fact that the information we collect is anonymous. It is because we avoid using devices that compromise the occupant's familiarity with the environment or make her/him feel monitored by wearing an RFID tag, for example (such systems also have a drawback if the occupant decides to withdraw the tag.). In this regard, our system is destined to be used to locate a single person in the house. When visitors are present, our system could be used in conjunction with another system: for example, requiring that visitors wear an RFID tag in order to differentiate them from the occupant. In this case, locating the occupant may also be regarded as less important than when the occupant is alone at home (DOMUS is designed for only one person with cognitive deficiencies per smart home. Visitors are mainly caregivers or family members).

In order to further evaluate our system, we study how it behaves regarding other experimental aspects, such as the analysis of possible correlations with the occupant's profile. We also investigate the best sensor configuration and their comparison per activity, the occupant's dynamics, the behavior in presence of noise, and finally the performance of the system.

### 7.1. Correlation with the occupant's profile

**Figure 5:**
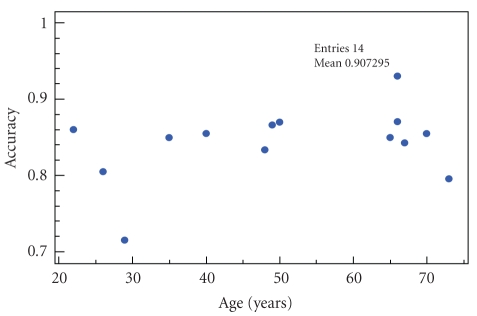
Variation of accuracy with the subjects' age.

One of the main objectives of our study is to evaluate whether or not the system should be personalized according to the occupant's profile. The only profile-dependent parameter that influences the Bayesian filtering formula is the dynamics. Since the subjects were healthy, without notable variations, the most accurate variable that relates to the dynamics is the age. Therefore, the hypothesis is that if the system depends on the occupant's age or on their dynamics, consequently on their profile, the system would require adjustments upon deployment to fit them. Figure 5 shows that there is no significant correlation between accuracy and age (correlation coefficient *ρ* = 0.36) and that accuracy remains stable from a subject to another. This result leads to two interesting conclusions. First, the data collected shows sufficient consistency to allow us to limit the number of subjects to 14. In fact, increasing this number will not give more confidence in the results. Second, it gives us the ability to deploy this system immediately in new homes, since it is profile-independent. This is good news regarding both economic and time constraints (obviously, a configuration phase is required for all deployments, and would be of approximately equal lengths given an apartment size and a required number of sensors).

### 7.2. Sensor configuration

In the purpose of addressing economic concerns, we study which sensor configurations are optimal. This information is important if one has to limit the redundancy, even though the latter could be useful to provide a robust environment. We therefore measure the system's accuracy using different sets of sensors. The complete information is in Table 3. In order to achieve this comparison, we use data from only one representative subject, whom we choose based on the mean age (50 ± 17 years old) and the duration of the experiment (48 ± 8 minutes). The values of the accuracy and mean belief using all the sensors are then slightly different than the mean ones presented in the previous section. The infrared sensors alone give comparable results with those obtained using all the sensors. That is due to the fact that their number (there is at least one such sensor in each room) and their spatial configuration (each one covers roughly the area of a room) dominate the global sensor configuration. However, it is worth noting that infrared sensors are not the most reliable type, for they are more prone to false activations, due to animal presence or a heat disturbance, for example. Consequently, deploying the system in a real setting would probably require at least one other type of sensors to ensure reliability.

### 7.3. Sensors per activity

The sensors are compared based on an activity criterion. We select two activities: walking and preparing a sandwich. Other activities—such as dining, reading and sleeping—do not generate enough sensor data with the present scenario to be considered for this comparison. Table 4 shows the results we obtain using different sets of sensors. For the walking activity, the IR sensors are the most accurate, because the occupant seldom activates other sensors while moving around the house. However, when preparing a sandwich, the occupant often opens the fridge and various drawers in the kitchen. Therefore, the door contact sensors are almost as accurate as the IR ones. This becomes useful for localization in case the activity being performed is known. In the previous section, we saw that IR sensors are at the core of this localization system, although they can present reliability issues. In case of preparing dinner, door contacts can be as accurate as and even more reliable than IR sensors, consequently more appropriate to locate the occupant during that activity.

### 7.4. The dynamic model

**Figure 6:**
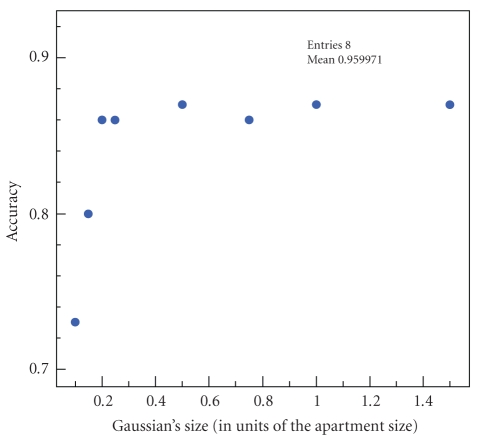
Variation of the system's accuracy with the occupant's dynamics, modeled by the Gaussian function's *σ*.

In order to test the effects of the dynamics on the model, we analyze the system's accuracy while modifying the Gaussian dynamic function. The system's accuracy remains stable on a long range of the Gaussian's size (Figure 6). However, when the standard deviation of the Gaussian becomes too small, the particle cloud inertia increases and the model fails to reflect a natural motion. On the contrary, larger functions seem to behave very well. But this changes when coping with noise.

### 7.5. Behavior in presence of noise

The experimentation took place in a laboratory. Therefore, even if the setting is similar to that of an apartment, disturbances are controlled. The subject is always alone and performing predefined activities. It is consequently important to analyze the behavior of the localization system in case of noise. First of all, since no significant noise was present in the data we collected, we generate random noise in order to complete our tests. Table 5 shows how the accuracy and mean belief are affected when contaminating the data with random sensor noise. The system is exceptionally stable and remains accurate at 84% even in the presence of 2.5% noise. Increasing the percentage of noise to 5% does not affect the localization accuracy. It is worth noting that since there was no observable noise in the data, 1% of noise is already a conservative value.

**Figure 7:**
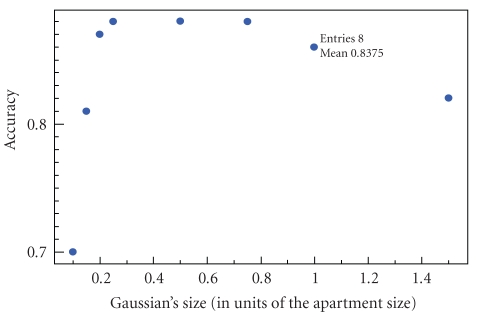
Variation of the system's accuracy with the dynamics, in presence of 2.5% noise.

In a second step, we analyze the impact of the noise when changing the dynamic model. We thus introduce 2.5% noise while modifying the dynamics (Figure 7). Small Gaussians still fail to reproduce the occupant's dynamics. However, in the presence of noise, larger Gaussians become problematic too. The larger Gaussians model faster motion, therefore failing to reject false events even if they are significanty distant than the actual occupant's location. The Gaussian's size has then to be comparable to that of the apartment to ensure the best system's accuracy.

### 7.6. The ideal number of particles

**Figure 8:**
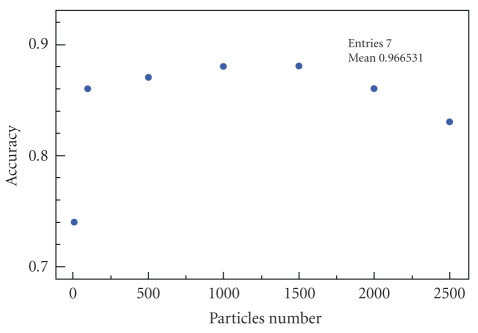
Variation of accuracy depending on the number of particles used in the algorithm.

Finally, for the sake of optimization, we want to infer the best parameters' settings for the algorithm. Therefore, we study how the number of particles used may affect the prediction accuracy (Figure 8). As expected, the more particles are used, the best the prediction is. However, an increase in the number of particles leads to an increase in computing time. Therefore, the cloud dynamics fails to replicate the occupant's motion. The system's accuracy is fairly stable when the particle number is in the range 500 to 2000. Therefore, the smallest value (500) becomes the best choice since it is the closest to reproduce the occupant's motion in real time.

## 8. CONCLUSION

We presented in this paper the localization system we put in place in the DOMUS laboratory. This system detects a single person's location by means of various anonymous sensors installed in the smart home. We set an experimental scenario in order to evaluate the accuracy, with people performing significant tasks in the smart home. The results obtained with these data are very satisfactory. The algorithm based on Bayesian filtering shows a mean localization accuracy of 85%. The system is fast and robust regarding noise. Moreover, since it is profile-independent, it can easily be deployed in the future homes that are being conceived in the laboratory. It will be interesting to observe the behavior of our system once it gets integrated in applications meant to provide high levels of assistance. Above all, it is crucial to measure the localization accuracy needed for different sets of applications. A possible continuation of this work would be to check the possibility to locate two or more people with the same experimental setup (the presence of pets is also interesting to investigate). This would enable to respect the cost and anonymousness constraints when several people are present. A multiagent approach is being investigated at DOMUS and already is giving promising results. Moreover, using data from people with cognitive deficiencies, in a real setting, would help in consolidating the results from the present study.

## Figures and Tables

**Table 1:** List of sensors per room.

	Entrance hall	Living room	Dining room	Kitchen	Bathroom	Bedroom	Total
IR	1	1	2	2	3	1	10
Tactile carpets	1	3	2	6	3	3	18
Light switches	1	1	1	3	1	1	8
Door contacts	3	0	0	30	5	10	48
Pressure detectors	0	0	0	0	0	1	1

**Table 2:** Density functions per sensor type.

Sensor	Density function type	Typical density function range (*m* ^2^)
IR	Square	20
Tactile carpets	Square	0.8
Light switches	Circular	1
Door contacts	Circular	1.5
Pressure detectors	Square	2

**Table 3:** Accuracy and mean belief using different sensors.

Sensor set	Accuracy (%)	Mean belief (%)
All	87	88
IR	88	89
Light switches	50	75
Light switches and door contacts	77	80

**Table 4:** Accuracy and mean belief per activity, using different sensors.

Activity	Sensor set	Accuracy (%)	Mean belief (%)
Walking	All	88	88
	IR	87	89
	Light switches	33	75
	Light switches and door contacts	38	72
Preparing sandwich	All	90	89
	IR	91	90
	Light switches	10	60
	Light switches and door contacts	85	87

**Table 5:** Variation of accuracy and mean belief with noise.

Noise (%)	Accuracy (%)	Mean belief(%)
0	88	88
1	88	88
2.5	84	85
5	84	85
